# Enhancing Targeted Genomic DNA Editing in Chicken Cells Using the CRISPR/Cas9 System

**DOI:** 10.1371/journal.pone.0169768

**Published:** 2017-01-09

**Authors:** Ling Wang, Likai Yang, Yijie Guo, Weili Du, Yajun Yin, Tao Zhang, Hongzhao Lu

**Affiliations:** 1 School of Biological Science and Engineering, Shaanxi SCI-TECH University, Hanzhong, Shaanxi, China; 2 Department of Pathogenic Microbiology and Immunology, School of Basic Medical Sciences, Xi'an Jitong University, Xi'an, Shaanxi, China; Qingdao Agricultural University, CHINA

## Abstract

The CRISPR/Cas9 system has enabled highly efficient genome targeted editing for various organisms. However, few studies have focused on CRISPR/Cas9 nuclease-mediated chicken genome editing compared with mammalian genomes. The current study combined CRISPR with yeast Rad52 (yRad52) to enhance targeted genomic DNA editing in chicken DF-1 cells. The efficiency of CRISPR/Cas9 nuclease-induced targeted mutations in the chicken genome was increased to 41.9% via the enrichment of the dual-reporter surrogate system. In addition, the combined effect of CRISPR nuclease and yRad52 dramatically increased the efficiency of the targeted substitution in the myostatin gene using 50-mer oligodeoxynucleotides (ssODN) as the donor DNA, resulting in a 36.7% editing efficiency after puromycin selection. Furthermore, based on the effect of yRad52, the frequency of exogenous gene integration in the chicken genome was more than 3-fold higher than that without yRad52. Collectively, these results suggest that ssODN is an ideal donor DNA for targeted substitution and that CRISPR/Cas9 combined with yRad52 significantly enhances chicken genome editing. These findings could be extensively applied in other organisms.

## Introduction

The chicken is one of the most important commercial animals and model organisms. Genetically modified chickens have great potential for application in fields such as agriculture, industry, biological research and pharmaceuticals. Targeted genomic DNA editing technologies are invaluable tools used to generate transgenic chickens to improve meat and egg quantity and quality, enhance disease resistance ability, and the produce protein drugs. Traditionally, transgenic chickens have been primarily generated using retrovirus vector-harboring target sequences. However, the virus vector-mediated target efficiency was low, and virus-induced side effects remained to be addressed. These issues slowed the development of transgenic chicken research and application. With the advent of artificial nucleases, the generation of genetically modified chickens is much more feasible than before. Designed nucleases can specifically induce DNA double-stranded breaks (DSBs). Subsequently, DSBs stimulate the cellular repair mechanisms of homologous recombination (HR) or non-homologous end joining (NHEJ). Thus, nuclease-mediated gene integration is much more precise and efficient than spontaneous recombination via donor DNA alone. In the absence of donor DNA, DSBs are repaired via NHEJ to induce small deletions or insertions (indels), which can lead to targeted gene disruption.

Zinc finger nucleases (ZFNs) were first assembled to target the 3' untranslated region of the chicken *ovalbumin* (*OVA*) gene in vitro but without the detection of a cleavage effect at genomic DNA *in vivo* [1]. In addition, transcription activator-like effector nucleases (TALENs) were designed to accomplish targeted knock out *OVA* gene in chicken primordial germ cells (PGCs), resulting in loss of chicken *OVA* gene function in offspring [2]. Recently, there is an unprecedented opportunity to edit genomic DNA with the clustered regularly interspaced short palindromic repeats (CRISPR) associated protein system, designated CRISPR/Cas system. Guided by single stranded RNA and Cas9 protein, CRISPR/Cas9 nucleases could target specific DNA sequences and generate DSBs in genomes [3]. Thus, exogenous genes or indels are accessibly introduced into chromosome DNA breaks, resulting in targeted transgenosis or mutagenesis [4,5]. Taking the advantage of highly efficient and specific DNA cleavage, CRISPR/Cas9 technology was used to successfully modify human cells at target sites precisely [6]. Compared with ZFNs and TALENs, CRISPR/Cas9 system revealed its accessible, versatile and precise merits for targeted genome editing in a large variety of species, such as mouse [7], rat [8], pig [9], goat [10], rabbit [11], zebrafish [12] and even plants [13,14].

CRISPR/Cas9 nucleases have been used extensively in chicken somatic and embryonic cells. Efficient gene disruptions of *PPAR-γ*, *ATP5E* and *OVA* were obtained in chicken somatic cells DF-1 with an enrichment system [15]. Véron *et al*. adopted CRISPR/Cas9 technology to disrupt the *Pax7* gene in chicken embryos [16]. In addition, chickens with targeted mutations in *OVA* and *ovomucoid* (*OVM*) were achieved via CRISPR/Cas9 nucleases, and these mutations were heritable [17]. Zou *et al*. demonstrated that CRISPR/Cas9-mediated gene knock-down efficiency was approximately 27% for both chicken DF-1 cells and embryonic stem cells (ESCs) [18]. Targeted knock-outs of the *JH* gene in chicken PGCs and gene-edited chickens were obtained via CRISPR/Cas9 nucleases [19]. Compared with human and mouse cells, the efficiency of customized gene editing in poultry and livestock is relatively low, which is the primary obstacle of developing transgenic animals in agriculture.

Yeast Rad52 (yRad52) is a key HR mediator that plays critical roles in Rad51 loading, DNA binding and single-stranded DNA (ssDNA) annealing during HR events [20]. Based on this mechanism, yRad52 was used to enhance gene targeting efficiency in mammalian cells to increase gene targeting by 37-fold via HR in Hela cells [21]. Furthermore, yRad52 fusion to the TAT protein of HIV (tat11) increased gene targeting up to 50-fold. In particular, the yRad52tat11 fusion protein maintained the ability to bind ssDNA to improve target integration efficiency and dramatically decrease random integration [22]. These findings suggest that yeast Rad52 can be used as a mediator to favor HR and decrease random integration for genetic modification. For this purpose, we designed yRad52 and Cas9 as a fusion protein using the CRISPR/Cas9 system to enhance target efficiency in which yRad52-mediated HR events occurred once Cas9 cut target DNA *in vivo*.

We employed the designed CRISPR/Cas9 nucleases to target *myostatin* (*MSTN*) and *Env* of endogenous avian virus (EAV-HP) in chicken DF-1 cells. In addition, a Cas9-fused yeast Rad52 protein was designed as a modified CRISPR/Cas9 system to mediate small nucleotide substitutions in chicken *MSTN* using single-stranded oligodeoxynucleotides (ssODN) as donor DNA. Furthermore, the EGFP expression cassette was targeted to insert into the EAV-HP *env* gene in DF-1 cells via this system. Therefore, we explored the possibility that the addition of yRad52 to the Cas9 protein enhances the effect in HR events and increase gene-targeting efficiency.

## Methods and Materials

### Cell culture and transfection

HEK293T and chicken DF-1 cell lines were used in this study. Cells were maintained at 37°C with 5% CO_2_ in Dulbecco’s modified Eagle medium (DMEM, Gibco, Beijing, China) supplemented with 10% fetal bovine serum (FBS, Gibco, Grand island, New York, the USA), 100 U/mL penicillin, and 100 mg/mL streptomycin. Plasmids were transfected into cells using SUPERFECT (Qiagen, Hilden, Germany). Transfection was conducted following the manufacturer’s instructions. Briefly, cells were seeded in 12-well plates before transfection. A mixture of plasmid DNA and transfection agents was added to the wells when the cell confluence reached approximately 60%. The cells were then maintained in an incubator.

### Construction of the CRISPR/Cas9 expression plasmid

**Fig 1:**
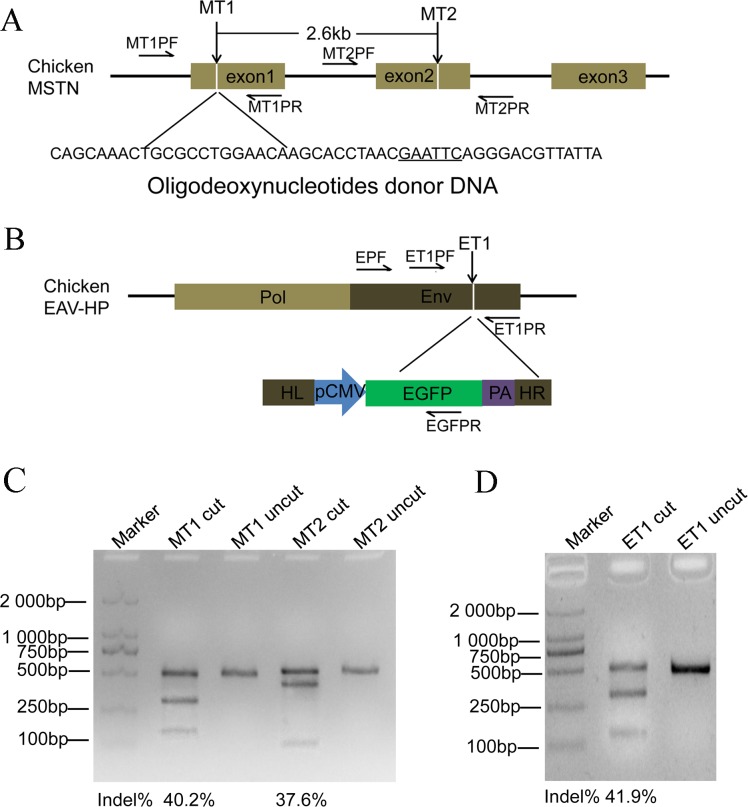
CRISPR/Cas9-mediated target mutagenesis in the chicken genome. (A) A schematic diagram of the target sites in the chicken *MSTN*. MT1 and MT2 were chosen as target sites in exons 1 and 2 of MSTN, respectively. The two sites span 2.6 kb in the genome. A 50-mer ssODN harboring an *Eco*RI site was designed to integrate the MT1 site. (B) A schematic illustration of the ET1 site in the chicken EAV-HP viral genome. One target site, ET1, was determined in the Env gene, and a relative donor DNA containing the *EGFP* expression cassette and two homology arms of *Env* was constructed for the exogenous gene knock-in of the chicken genome. HL and HR: left and right homologous arms; pCMV: CMV promoter; PA: polyA. (C) The efficiencies of mutagenesis in the MT1 and MT2 sites of the chicken genome via the T7 EI assay. (D) The efficiency of the targeted mutation in the ET1 site of the chicken genome using the T7 EI assay. DF-1 cells were co-transfected with the CRISPR/Cas9 expression plasmid and the corresponding reporter vector. The target sequence was inserted into two homology repeats to disrupt *Puro*^*R*^ and *eGFP* genes in the reporter vector. When Cas9 cut the target sequence in the reporter vector to generate a DSB, *Puro*^*R*^ was restored via SSA. The target sequences in both the reporter and chicken genomes were simultaneously cut via CRISPR/Cas9. Thus, puromycin was added to the cell culture to enrich the gene-editing positive cells. Targeted mutations in the chicken genome not in reporter vectors were detected using the T7 EI assay.

Obeying the NGG PAM sequence rule, two target sites, MT1 and MT2, were designed in exon1 and exon2 of chicken *MSTN* via a convenient online tool (http://crispr.mit.edu/), respectively (Fig 1A). An ET1 target site was chosen in the envelope gene of EAV-HP (Fig 1B). The three targets are shown in Table 1 and the corresponding primers for sgRNA construction are displayed in Table A in S1 Text.

**Table 1:** Target site sequences.

Target site	Sequences (5’- 3’)
MT1	ATAACGTCCCTGCTAATGTT AGG
MT2	GCTGTCACATTCCCAGGACC GGG
ET1	TACTCCTGGCGCAGGGACAC GGG

Note: Underlining indicates PAM sequences.

DNA fragments harboring chicken MSTN and EAV target sequences were generated via overlap PCR. For target MT1, two short DNA fragments were amplified via the primer pairs U6F/MT1R and MT1F/U6R (Table A in S1 Text), in which the two PCR products both had MT1 target sequences as overlap regions. Then, two short fragments were ligated together via overlap PCR and cloned into the parent plasmid pll3.7-U6-sgRNA-Cas9 between the *Xba*I and *Not*I sites, thereby resulting in the MT1 CRISPR/Cas9 expression vector designated pll3.7-U6-MT1sgRNA-Cas9. Moreover, the CRISPR/Cas9 vectors for MT2 and ET1 were obtained using the same strategy and designated pll3.7-U6-MT2sgRNA-Cas9 and pll3.7-U6-ET1sgRNA-Cas9. The sgRNA sequencing of the expression plasmids was performed using the U6F primer.

Another CRISPR/Cas9 nuclease expression vector was initially designed that expressed yeast Rad52 and Cas9 as a fusion protein. Yeast Rad52 was amplified via the primers yRadF and yRadR (Table A in S1 Text) from the yeast genome. The forward and reverse primers containing *Bsa*I sites and the same sticky ends with *Nhe*I and *Bam*HI were generated via the *Bsa*I digestion of the PCR products. Then, the yRad52 fragments were cloned into plasmid pll3.7-U6-sgRNA-Cas9 between *Nhe*I and *Bam*HI, thereby resulting in the yRad52-Cas9 fusion protein vector pll3.7-U6-sgRNA-yRad-Cas9. Subsequently, ET1 and MT1 target sequences were obtained via the double digestion of pll3.7-U6-ET1sgRNA-Cas9 and pll3.7-U6- MT1sgRNA-Cas9 and then subcloned into plasmid pll3.7-U6-sgRNA-yRad-Cas9 *Xba*I/*Not*I sites, thereby resulting in two plasmids, pll3.7-U6-ET1-yRad52-Cas9 and pll3.7-U6-MT1-yRad52-Cas9, respectively.

### Construction of reporter plasmid

The construction of reporter vectors was described previously [23]. Plasmid pCAG-puro-EGFP was used as the reporter vector for validating nuclease activities and screening positive cells with targeted gene editing (S1 Fig). Two complementary oligonucleotides harboring target and PAM sequences were synthesized for chicken MT1, MT2 and ET1 sites (Table A in S1 Text). Subsequently, target sequences with *Bam*HI and *Not*I sticky ends were generated via oligonucleotide direct annealing at 70°C for 10 min, then cloned into the *Bam*HI/*Not*I sites of the backbone vector pCAG-puro-EGFP, thereby resulting in the MSTN and EAV reporter plasmids pCAG-puro-MT1-EGFP, pCAG-puro-MT2-EGFP, and pCAG-puro-ET1-EGFP.

### Construction of donor DNA

Two DNA donors were designed for this study. For the ET1 target site, the plasmid donor DNA contained the EGFP expression cassette flanking two homologous arms with chicken EAV-HP genomic DNA (Fig 1B). The DNA fragment of the *EGFP* expression cassette was amplified from plasmid pEGFP-C1 using the primers EGFPF and EGFPR. The left and right homology arms were amplified from DF-1 genomic DNA via the primer pairs ELF/ELR and ERF/ERR, respectively. The PCR products of the left and right homologous arms and the EGFP expression cassette were ligated together via overlap PCR. Subsequently, the PCR products were purified and cloned into the pUC19 vector between the *Hin*dIII and *Eco*RI sites, thereby resulting in the recombinant plasmid pUC19-EGFP. The sequencing of pUC19-EGFP was performed to confirm the donor DNA sequences.

In addition, the donor DNA for the MT1 site was a 50-mer ssODN that spanned the MT1 target site and 5-point mutations to create an EcoRI site (GAATTC). The oligonucleotides were synthesized at a commercial company (AuGCT, Beijing, China). The primers and ssODN are displayed in Table B in S1 Text.

### Validation of CRISPR/Cas9 nuclease activity

The activities of the designed CRISPR/Cas9 nucleases were detected in HEK293T cells before use in DF-1 cells. HEK293T cells were seeded into 12-well plates, and the transformation was conducted when the cell confluence was approximately 60%. The CRISPR/Cas9 expression plasmids and their corresponding reporter plasmids or donor DNA were mixed with SUPERFECT agents thoroughly and added to the cell culture. Transformation with empty expression vectors and reporter plasmids were used as a control. The GFP positive cells were observed via fluorescence microscopy (Leica AF6000, Germany).

### CRISPR/Cas9-mediated targeted mutagenesis

DF-1 cells were transformed with 1 μg of sgRNA/Cas9 nuclease expression plasmids and 1 μg of their corresponding reporter vectors. Puromycin was added to the cell culture medium after the 48-h transformation, and selection was maintained for 4 days. After puromycin selection, the cells in each well were divided into two groups. One group was used to calculate the survival rate using propidium iodide (PI) staining as previously described [24]. The other group was grown in fresh medium without puromycin for 2~3 days and genomic DNA was extracted for further mutation detection.

Subsequently, DNA fragments containing target sites were amplified from genomic DNA with primer sets (Table C in S1 Text) and purified via gel extraction. The target sequences MT1 and MT2 were amplified via the primer pairs MT1PF/MT1PR and MT2PF/MT2PR, respectively. For the target ET1, PCR was performed via the primers ET1PF and ET1PR. The amplification was conducted with Taq polymerase via a touch-down PCR program as follows: 95°C for 5 min for initial denaturation; 16 cycles of touch-down amplification, consisting of 95°C for 30 s, 68°C for 30 s with 1°C reduction every cycle, 72°C for 30 s; 25 cycles of 95°C for 30 s, 52°C for 30 s and 72°C for 30 s; an additional extension at 72°C for 5 min; and 8°C hold.

To evaluate mutation efficiency, the PCR products were detected using the mismatch-sensitive T7 endonuclease I assay. First, 200 ng of the PCR products were denatured at 95°C and allowed to anneal gradually at room temperature to form heteroduplex DNA, which was digested with T7E I and resolved in a 2% agarose gel. The targeted mutation efficiency was determined using previously described methods. In addition, the PCR products were cloned into plasmid pMD19-T for sequencing using the universal primer M13F (-47).

### CRISPR/Cas9-mediated targeted substitution in the MSTN gene

Cells transfected with 0.5 nmol ssODN only were used as the control. In addition to ssODN, four experimental groups were designed to treat cells with different plasmids and DNA. Group 1 cells were transfected with 0.5 nmol ssODN and 1 μg pll3.7-U6-MT1sgRNA-Cas9, and the cells in group 2 were treated with ssODN, pll3.7-U6-MT1sgRNA-Cas9 and 1 μg reporter vector pCAG-puro-MT1-EGFP. In addition, DF-1 cells transfected with ssODN and pll3.7-U6-MT1sgRNA-yRad-Cas9 were used as experimental group 3. The cells in group 4 were transfected with ssODN, pll3.7-U6-MT1sgRNA-yRad-Cas9 and 1 μg pCAG-puro-MT1-EGFP. Cells treated with reporter vectors were selected using puromycin to enrich positive cells. After puromycin selection, the survival rate was determined via PI staining.

Cells were harvested, and genomic DNA was extracted via the phenol-chloroform method. Then, the genomic DNA of MT1 was amplified with the MT1PF and MT1PR primers via touch-down PCR. The PCR products were purified via gel extraction, digested with *Eco*RI, and detected on 2% agarose gel. The proportion of cleaved DNA was quantified using densitometry, and sequencing was performed to confirm the targeted integration of the *Eco*RI site.

### CRISPR/Cas9-mediated targeted knock-in at the ET1 site

Exactly 1 μg of ET1 nuclease expression vectors and 1 μg donor plasmid pUC19-EGFP were co-transfected into DF-1. Cells transfected with 1 μg of donor plasmids were used as controls. EGFP-positive cells were observed under fluorescence microscopy. Cells were passaged into 24-well plates every two days. After two weeks, EGFP expression in the cells was detected via fluorescence microscopy in each well, and EGFP-positive cells were determined using flow cytometry (Partec Cube6, Germany). In addition, EGFP-positive clones were separated via limited dilution and transferred to 96-well plates. The genomic DNA was isolated from the cell pools after 2 weeks. PCR was performed to amplify the integrated EGFP gene in the DF-1 chromosome using the following cycling condition: 95°C for 5 min for initial denaturation; 30 cycles at 95°C for 30 s, 53°C for 30 s and 72°C for 1 min and 30 s; and a final extension at 72°C for 10 min. The primers EPF and EGFPR bound to the EAV genomic region, and EGFP gene sequences were used to amplify specific DNA fragments to confirm that the EGFP expression cassette integrated into the ET1 site successfully. The sequencing of the PCR products was performed to further verify EGFP-targeted insertion.

### Off-target analysis

To investigate the off-target effect in the chicken genome, potential off-target sites were analyzed for each target site via the screening of the whole chicken genome with the online tool found at http://crispr.mit.edu/. Then, two candidate off-target sites with the highest homology scores were chosen for the MT1 site, and three potential off-target sites were identified for the MT2 and ET1 sites, respectively (Table B in S2 Text). Off-target sequences were amplified via touch-down PCR from the sgRNA-treated cell genomes using the primers listed in Table B in S2 Text. The T7E I assay was used to measure the off-target effect of these potential targets.

## Results

### CRISPR/Cas9 nuclease-mediated targeted mutagenesis

A dual-reporter surrogate system was used to validate nuclease activities and enrich gene-editing positive cells in a previous study [23]. The current study used this system and designed a reporter vector for each target of MT1, MT2 and ET1. To determine whether the designed sgRNA could guide Cas9 nuclease to cut target sequences, the three CRISPR/Cas9 expression vectors and their reporter plasmids were co-transfected into HEK293T cells. After CRISPR/Cas9-induced DSBs at target sites in the puromycin resistance gene (*Puro*^*R*^) were repaired via SSA, the reporter genes *Puro*^*R*^ and *GFP* were restored to generate GFP-positive cells. In fact, two 200 bp homology repeats, in which the open reading frame (ORF) of wild-type *GFP* was disrupted, separated the *puro*^*R*^ gene. Once the *puro*^*R*^ gene was repaired, the ORF of *GFP* was also correct. Thus, either puromycin selection or GFP-positive selection indicated the nuclease cleavage efficiency (S1 Fig). As such, GFP expression was readily detected in HEK293T cells to verify the cleavage efficiency of the designed CRISPR/Cas9 nucleases.

Subsequently, the co-transfection of the CRISPR/Cas9 nuclease vectors and their corresponding reporter vectors into DF-1 cells was conducted for each target MT1, MT2 and ET1 site (Fig 1A and 1B). Moreover, cells transfected with empty vectors were used as controls. To enrich cells with targeted mutations, puromycin was added into the medium at after the 48-h transfection and maintained for 96 h. The cell survival in the experimental groups was higher than that in controls (S2 Fig). The genomic DNA was isolated from surviving cells. The PCR products of target sequences in the chicken genome were purified, and the T7 EI assay was used to determine mutation efficiency. Based on the puromycin selection, the mutation efficiencies of the MT1 and MT2 in the chicken genome were 40.2% and 37.6% (Fig 1C), respectively. For the target ET1, the efficiency was approximately 41.9% (Fig 1D). However, targeted mutations were nearly undetectable without puromycin enrichment.

**Fig 2:**
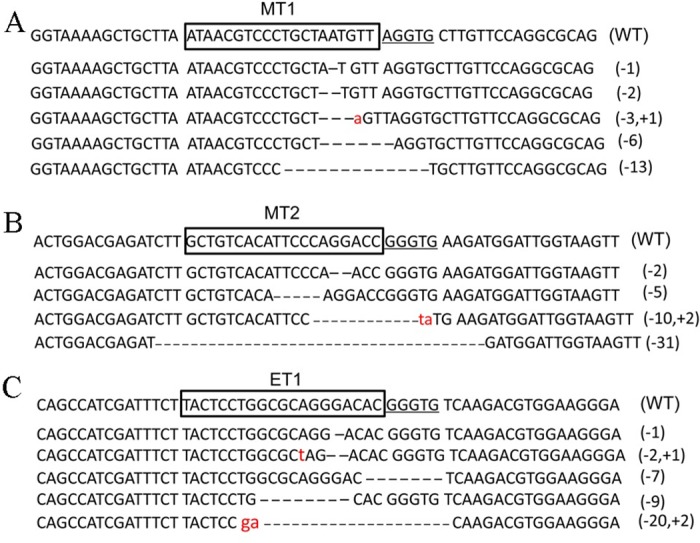
CRISPR/Cas9-induced mutations of the three target sites in the chicken genome. (A) The sequencing results of the indels at the MT1 site. (B) The sequencing results of the indels at the MT2 site. (C) The sequencing results of indels at the ET1 site. WT: wild-type genome sequence. Small deletions and insertions in the sequences are marked with negative and positive numbers.

In addition, PCR products were cloned into pMD19-T, and 10 colonies were chosen for sequencing. For the MT1 site, indels were observed in 5 colonies, in which the deletion of 1 to 13 nucleotides occurred at MT1 target sites. One nucleotide insertion was also detected at the Cas9-cleavage positions of MT1 (Fig 2A). Only 4 mutations were gained from the 10 colonies for MT2, in which small deletions from 2 to 31 nucleotides were displayed (Fig 2B). A 2-nucleotide insertion was also observed at the event of a 10-nucleotide deletion. Five of ten colonies revealed indel mutations at ET1 sites. Deletions in the ET1 target or PAM sequences were detectable, and an additional one- or two-nucleotide insertion occurred during NHEJ repair (Fig 2C).

### CRISPR/Cas9 nucleases generated a large fragment deletion

**Fig 3:**
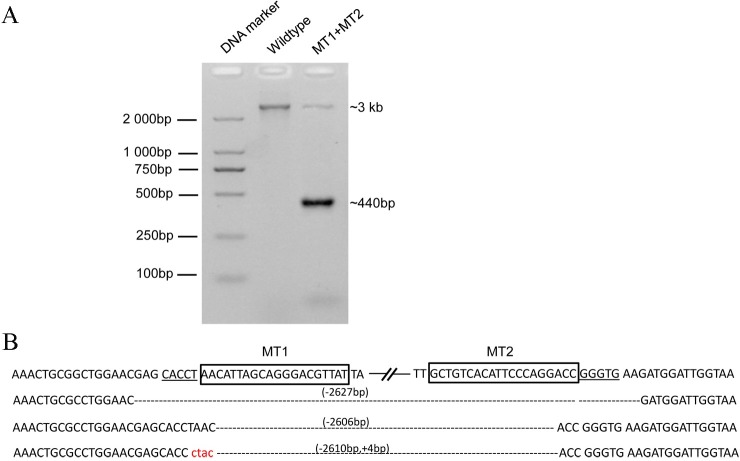
CRISPR/Cas9-induced large fragment deletions in the *MSTN* gene. (A) PCR amplification of the DNA fragments spanning the MT1 and MT2 sites. Only the 3-kb fragment was obtained when wild-type genomic DNA was used as the template. Thus, another 440-bp fragment was achieved from both the MT1 and MT2 CRISPR/Cas9-treated cells. (B) The sequencing results of the deletion events.

The distance between MT1 and MT2 is approximately 2.6 kb (Fig 1A). To detect the large fragment deletion efficiency in chicken chromosomes, two sgRNA expression vectors, MT1 and MT2, and their reporter vectors were co-transfected into DF-1 cells. After puromycin selection, genomic DNA was extracted from surviving cells. The DNA fragments of expanded MT1 and MT2 were amplified from genomic DNA via the primers MT1PF and MT2PR. Only 3.0 kb fragments were obtained from wild type genomic DNA templates, whereas two DNA fragments of approximately 3.0 kb and 440 bp were observed from the cells treated with both MT1 and MT2 CRISPR/Cas9 (Fig 3A). These data indicate that the two sgRNAs cut the chicken MSTN gene simultaneously to generate a 2.6 kb fragment deletion.

Next, 440 bp of PCR products were cloned into the pMD19-T vector for sequencing using the primer M13 (-47). The sequencing results revealed that the sequences between the two sites were completely removed from the genome. The 2,627-bp deletion in the chicken chromosome included the two target sites and PAM sequences. A 2,602-bp deletion was present between the Cas9-cleavage sites in both target sites. In addition, the insertion of 4 nucleotides was observed in the 2,610-bp deletion event (Fig 3B).

### CRISPR/Cas9 enhanced targeted genome editing

**Fig 4:**
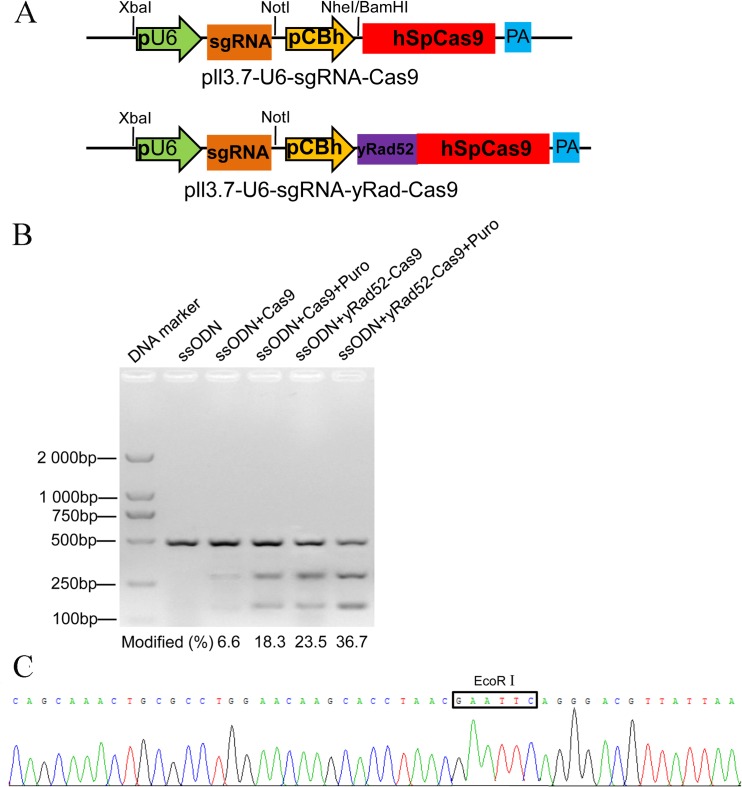
CRISPR/yRad52-Cas9 enhances targeted substitutions via ssODN at the MT1 site. (A) A schematic diagram of the CRISPR/Cas9 and CRISPR/yRad52-Cas9 expression vectors. (B) The frequency of ssODN substitution in the genome of DF-1 cells after different treatments. After the co-transfection of reporter and expression vectors into the DF-1 cells, CRISPR/Cas9 simultaneously cut target sites in both reporter vectors and the genome. (C) The DNA sequence of the genome modified with EcoRI integration at the MT1 locus. Letters marked with a pane are the *Eco*RI enzyme recognition sequence.

Donor DNA with homologous sequences was integrated into the genome during the HR repair event. Homology ssODN was also used as donor DNA to accomplish targeted nucleotide substitutions. The current research designed ssODN containing an EcoRI site flanked by 50 nt of homology on the MT1 cut site (Fig 1A). The CRISPR/yRad52-Cas9 expression vector was constructed to investigate whether yeast Rad52 efficiently enhances ssODN insertion into the MT1 locus (Fig 4A). Then, ssODN with MT1 CRISPR/Cas9 expression and reporter vectors were co-transfected into DF-1 cells. Moreover, DF-1 cells transfected with ssODN only were used as controls. Puromycin selection was conducted to enrich positive cells (S3 Fig). MT1 sequences were amplified from treated cell genomes, and the PCR products were digested with EcoRI to determine their integration efficiency. In the control cells, ssODN integration was undetectable. By contrast, the integration efficiency of the cells treated with ssODN and plasmid pll3.7-U6-MT1sgRNA-Cas9 was approximately 6.6%. The efficiency of the yRad52-Cas9 fusion protein-mediated ssODN insertion was 23.5%. Without puromycin selection, yRad52-Cas9 improved the efficiency of the ssODN insertion approximately 4-fold compared with Cas9. The additional puromycin selection of the cells transfected with reporter vector revealed that the efficiencies of Cas9- and yRad52-Cas9-induced specific integration were approximately 18.3% and 36.7%, respectively (Fig 4B). Thus, the yRad52-Cas9-mediated integration rate was twice the Cas9-mediated integration rate. These sequencing results further confirm the integration of the EcoRI sequence at the MT1 site (Fig 4C).

### CRISPR/Cas9 improved targeted exogenous gene knock-in

**Fig 5:**
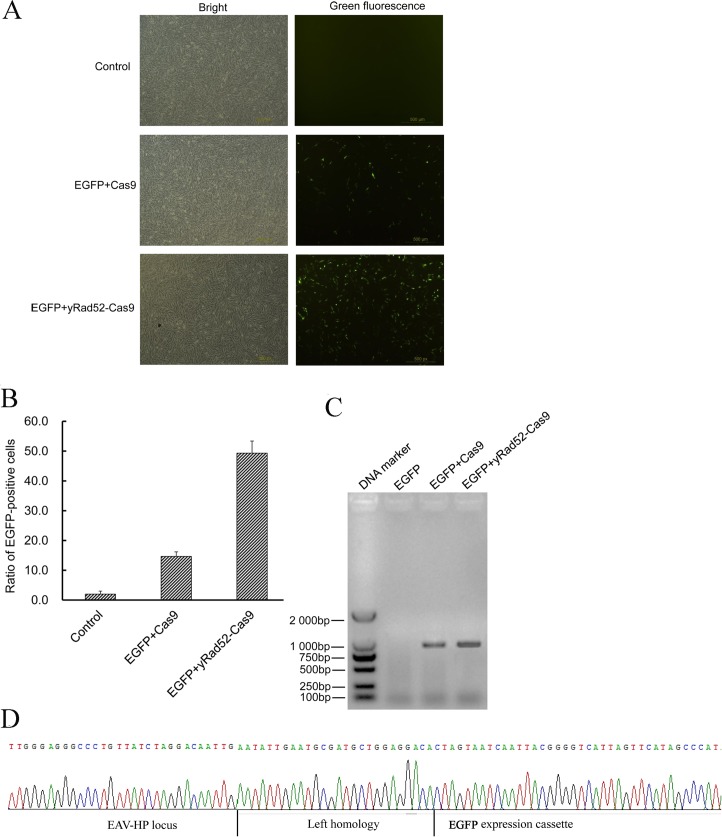
CRISPR/yRad52-Cas9 improves targeted knock-ins at the ET1 locus. (A) EGFP-positive cells using fluorescence microscopy. EGFP expression in DF-1 cells was originally checked via fluorescence microscopy after continuous passage for two weeks. Scale bar = 500 μm. (B) The determination of EGFP-positive cell ratio via flow cytometry. (C) The detection of *EGFP* integration in the chicken genome via PCR. PCR was performed to amplify DNA fragments spanning the EAV-HP viral genome and the *EGFP* gene from treated cell genomes. (D) The DNA sequencing of integrated *EGFP* at the ET1 site in EGFP-positive clones. Partially integrated EGFP expression cassette, left homology and EAV-HP genome.

EAV-HP virus, an endogenous avian virus, was integrated into the chicken genome during evolution and stably transferred across generations. Viral gene expression has no obvious effects in chickens. Thus, we assumed that the EAV-HP virus genome was a safe harbor for exogenous gene knock-ins of the chicken chromosome with the hope that the genetic modification would stably transfer to the next generation. Hereby, we transfected donor plasmids of the pUC19-EGFP and ET1 CRISPR/Cas9 expression vectors into DF-1 cells. Fig 1B shows the construct of donor EGFP. Cells transfected with pUC19-EGFP only were used as a control. EGFP-positive cells were observed via fluorescence microscopy. Then, continuous cell passage for 2 weeks was performed to detect stable *EGFP* gene expression (Fig 5A).

By comparison, the ratio of EGFP-positive cells in wells treated with the yRad52-Cas9 fusion protein was 49.3%, which was far higher than the 14.7% positive cells transfected with pll3.7-U6-ET1sgRNA-Cas9 (Fig 5B). To further confirm that the EGFP expression cassette integrated into the chicken chromosome, PCR was conducted to detect the EGFP gene in treated cell genomes with the primers EPF and EGFPR (Table C in S1 Text). The primer EPF was positioned at the EAV-HP genome but outside of the homologous left arm sequences; however, the EGFPR primer targeted the exogenous EGFP gene. No specific fragments were obtained from control cell genomic DNA. The expected 1,200 bp fragments were isolated from cells treated with donor plasmid and the CRISPR/Cas9 expression vector (Fig 5C). Furthermore, PCR sequencing results verified EGFP-targeted integration into the ET1 locus (Fig 5D).

### Off-target analysis

Previous studies have observed CRISPR/Cas9-induced off-target effects in multiple mammalian cells. To reveal the off-target effects of sgRNA in chicken cells, we analyzed the potential off-target sites in genomes from cell pools treated with CRISPR/Cas9 nucleases. Two and three candidates were chosen for the MT1 and MT2 sites, respectively (Table A in S2 Text), and three candidate off-target sites were chosen for ET1. These sequences were amplified and analyzed using the T7E1 assay. Only the OT2-MT1 candidate displayed 2.8% off-target efficiency with MT1 CRISPR/Cas9 nucleases, which covered 16 nucleotides followed by the AAG PAM sequence. However, no obvious off-target effects were observed for the other 7 candidates (S4 Fig).

## Discussion

As a member of the transforming growth factor beta (TGF-β) superfamily, MSTN is a negative regulator of skeletal muscle growth and is highly conserved among animals [25]. Previous reports have suggested that natural MSTN mutations cause hypermuscularity in cattle [26] and sheep [27]. In addition, genetically knocked-out pig and sheep *MSTN* demonstrated a double-muscled phenotype and increased muscle mass [28,29]. In addition, mutations on *MSTN* exon 1 significantly affect the growth traits of Bian chickens [30]. Thus, *MSTN* mutations or disruption can also lead to increases in chicken muscle. The current paper modified the chicken *MSTN* gene via the CRISPR/Cas9 system to obtain *MSTN* knock-out chicken cells. The modified efficiency was 40.2% for a single site. Moreover, two sgRNA targets, exon1 and exon2, were used to treat chicken genomes to generate a 2.6-kb chromosome deletion, which was a far more complete knock-out than disruption at a single site. This strategy provides a feasible tool for chicken germline cells in further study.

Targeted gene editing is more accessible when customized nucleases induce DSBs in genomes. The issue of enriching positive cells with genetic modifications has yet to be addressed. Many studies have focused on the development of efficient selection approaches for enriching genetically modified cells. To achieve this goal, surrogate reporter vectors harboring target sites were constructed to mimic target sequences in chromosomes. In principal, the episomal reporter gene containing the target sequence should faithfully reflect the nuclease’s activity on the chromosome in the same cell. Kim’s research team established several enrichment systems based on magnetic separation, antibiotic selection and fluorescence-activated cell sorting (FACS), which have been used successfully to enrich cells with targeted gene mutations [31–33]. The current study used a dual reporter system as previously described to validate nuclease activities as well enrich cells with nuclease-induced mutations [23]. This surrogate system was based on using the puromycin resistance gene and *EGFP* as double reporter genes to enrich cells with mutations induced by CRISPR/Cas9 nucleases in the human and chicken genomes. In contrast, the mutation frequency of chicken *PPAR-γ* was 60.7% under puromycin selection; however, only a 0.75% mutation efficiency was detected without puromycin selection [15]. Based on this finding, we directly used this reporter system to enrich cells with targeted mutations. After puromycin selection, nuclease-induced modification efficiencies were 40.2%, 37.6% and 41.9% for the MT1, MT2 and ET1 sites, respectively. By compared with the previous studies, the efficiency of mutation in chicken cells is not so high (41.9%) with the help of reporter system. However, without reporter system, the mutation was rarely detectable in this study. We speculated that mycoplasma contamination in cell culture led to relatively low editing efficiency. Even though, we added ciprofloxacin into cell culture to inhibit mycoplasma infection, which also decreased the effect of CRISPR/Cas9 system. In addition, we used this reporter system to enrich cells with targeted substitutions via ssODN donor DNA.

Donor DNA with homologous sequences is essential for targeted exogenous gene insertions or small nucleotide substitutions. Typically, the construction of donor plasmids containing 200–800 bp homology arms flanking the genome target sites requires several weeks [34]. By contrast, ssODNs can be easily designed and synthesized in a few days, which benefits gene targeting and substitution as donor DNA. Chen *et al*. systematically determined that the efficiency of ssODN donor-mediated genome editing depends on cell type and homology length [35]. That study demonstrated that the insertion efficiency ranged from 7% to 57% across different cell types and that the efficiency dramatically decreased when the base homology transitioned from 40-mer to 30-mer. And 50-mer homology mediated insertion efficiency was much higher than 40-mer. But efficiencies were not obviously increasing when homology up to 60, 70, 80, 90 or even 100-mer. Basing on this finding, we chose 50-mer homology ssODN as donor DNA. Subsequently, ssODNs combined with CRISPR/Cas9 nucleases were used to generate precise point mutations and defined editing in the genomes of mice [36] and plants [37]. Based on these findings, the CRISPR/Cas9 system was extensively used to insert small nucleotides into chicken MSTN with ssODN as the donor DNA. With 50 total bases of homology, CRISPR/Cas9-induced ssODN insertion efficiency increased from 6.6% to 18.3% in the presence of puromycin selection. Chen *et al*. also found that the single-stranded format of oligodeoxynucleotides resulted in fewer nonfaithful integrations than a double-stranded oligodeoxynucleotide composed of the same ssODN, but we did not detect this point in the current study. Thus, this finding provides an efficient strategy to introduce customized mutations in chicken *MSTN* gene in PGCs for further study.

Because of the differences in cell types, the ssODN insertion efficiency in this study was much lower than that previously described in human cells [35]. Several efforts have been made to increase the frequency of targeted knock-in over random integration in the genome such as increasing the length of homology between donor DNA and the target locus [35], inducing a DSB at the target site, or overexpressing recombination proteins [2,38]. Even inhibiting the expression of the proteins involved in non-HR was used to enhance targeted knock-in via RNA interference. We hypothesized that inducing DSBs combined with the overexpression of combination proteins would dramatically enhance gene targeting. To achieve this goal, we combined yeast Rad52 and Cas9 nuclease to form a fusion protein using this system.

As a critical recombination mediator, yRAD52 was involved in HR events to repair DNA DSBs in *Saccharomyces cerevisiae* [39]. The overexpression of yRad52 might switch the balance toward HR to enhance gene-targeting efficiency by 37-fold in Hela cells. Furthermore, the yRad52-tat11 fusion protein was designed to increase the frequency of gene targeting over random integration [22]. Inspired by these findings, we fused the yRad52 coding sequence to the Cas9 guided by sgRNA to cleave DNA at target sites. We hypothesized that once Cas9 induced DSBs at designed sites, yRad52 carrying ssDNA would trigger the HR mechanism to immediately repair DNA breaks. We found that yRad52-Cas9-mediated ssODN targeted insertion increased to 23.5%, whereas the insertion efficiency was only 6.6% without yRad52. Moreover, yRad52-Cas9 enhanced *EGFP* integration into the ET1 site by 3-fold compared with that without yRad52.

EAV-HP is an EAV retrovirus family that exists as a stable genetic element in the chicken genome. In the course of co-evolution with chickens, most EAV-HP proviruses underwent large sequence deletions, including the entire *pol* gene, thereby resulting in defective elements [40]. However, some evidence indicates that EAV-HP led to the emergence of avian leukosis virus subgroup J, which caused severe infection in chickens worldwide [41]. Because of the numerous point mutations, deletions and insertions in the sequences, EAV-HP is likely a helper virus that inactivates viral gene products. To date, none of the proviruses have been observed to produce infectious virions [42]. Thus, we assume that the EAV-HP genome is a safe harbor for exogenous gene integration that has little effect on functional gene expression in the chicken genome. Consequently, the *EGFP* expression cassette initially attempted to knock-in the *env* gene of the EAV-HP genome via CRISPR/Cas9. By contrast, the yRad52-Cas9-mediated integration efficiency was much higher than the Cas9-induced target rate. However, the safety of this target site must continue to be validated in future studies.

In summary, we combined the CRISPR/Cas9 nuclease with yRad52 to create a more robust tool for targeted genome editing in chicken cells. This novel approach can be used extensively in other organisms and increase the accessibility of gene modification in poultry and livestock.

## Supporting Information

S1 Fig Validation of CRISPR/Cas9 nuclease activities via the reporter system. (A) A schematic diagram of the dual reporter surrogate system. Under the control of the CMV promoter, the expression of DsRed was directly detectable after transfection into HEK293T cells. Two direct repeats and target sequences with PAM divided the puromycin resistance gene. A customized CRISPR/Cas9 cut at the target sites in the reporter vector to generate DSBs, which were repaired via single strand annealing in the presence of homologous arms. Subsequently, the wild-type puromycin resistance (*puro*^*R*^) gene was restored, as was functional eGFP. Thus, *puro*^*R*^ and *eGFP*, as dual reporter genes, were used for CRISPR/Cas9 activity validation and gene-editing positive colony screening. (B) Validating the targeted cleavage of designed CRISPR/Cas9 in HEK293T cells. CRISPR/Cas9 expression vectors and their corresponding reporter vectors at the MT1, MT2 and ET1 sites were co-transfected into HEK293T cells, respectively. Cells transfected with empty expression and nonsense reporter vectors were used as controls. RFP-positive or GFP-positive cells were observed via fluorescence microscopy. Scale bar = 200 μm.
(TIF)

S2 Fig The enrichment of cells with targeted modification via puromycin selection. DF-1 cells were co-transfected with CRISPR/Cas9 expression and their corresponding vectors. Puromycin was added to the cell culture medium after the 48-h transformation at a final concentration of 2.5 μg/mL. After selection for 4 days, cells were observed via microscopy, and the rates of survival were determined via flow cytometry. Cells transfected with empty CRISPR/Cas9 expression vector and reporter plasmid without target sites were used as controls. MT1, MT2 and ET1 indicate the related experimental groups of cells treated with vectors for the single target site. MT1+MT2 indicate cells treated with vectors for both the MT1 and MT2 target sites. After puromycin selection, the cells in each well were divided into two groups. (A) One group was maintained in fresh medium for observation via microscopy. Scale bar = 200 μm. (B) The other group was used to calculate the survival rate via PI staining.
(TIF)

S3 Fig Enrichment of cells containing ssODN targeted insertion at the MT1 site with puromycin selection. Cells transfected with 0.5 nmol of ssODN were used as controls. Cells treated with 0.5 nmol ssODN, 1 μg of MT1 reporter vector and 1 μg of MT1-CRISPR/Cas9 or CRISPR/yRad52-Cas9 expression vector were used as experimental groups 2 and 4. After transfection for 2 days, cells were maintained in medium with puromycin (2.5 μg/mL) for 4 days. After puromycin selection, cells were divided into two groups. (A) One group of cells was grown in fresh medium without puromycin for 2–3 days. The cells were then observed via microscopy. Scale bar = 200 μm. (B) The other cells were used to calculate the survival rate via PI staining.
(TIF)

S4 Fig Measurement of off-target effect on the potential candidates in chicken genomes. (A) The off-target effect of the two candidates for the MT1 target site. (B) The off-target effect of the three candidates for the MT2 target site. (C) The off-target effect of the three candidates for the ET1 target site.
(TIF)

S1 Text Primers for vector construction and the detection of targeted modification. (DOC)

S2 Text Potential off-target sites and their corresponding primers. (DOC)

## References

[1] Fan B, Huang P, Zheng S, Sun Y, Fang C, Sun Z. Assembly and in vitro functional analysis of zinc finger nuclease specific to the 3' untranslated region of chicken ovalbumin gene. Anim Biotechnol. 2011;22: 211–222. 10.1080/10495398.2011.626885 22132814

[2] Park TS, Lee HJ, Kim KH, Kim J-S, Han JY. Targeted gene knockout in chickens mediated by TALENs. Proc Natl Acad Sci U S A. 2014;111: 12716–12721. 10.1073/pnas.1410555111 25139993 PMC4156757

[3] Doudna JA, Charpentier E. Genome editing. The new frontier of genome engineering with CRISPR-Cas9. Science. 2014;346: 1258096 10.1126/science.1258096 25430774

[4] Ran FA, Hsu PD, Wright J, Agarwala V, Scott DA, Zhang F. Genome engineering using the CRISPR-Cas9 system. Nat Protoc. 2013;8: 2281–2308. 10.1038/nprot.2013.143 24157548 PMC3969860

[5] Jinek M, Chylinski K, Fonfara I, Hauer M, Doudna JA, Charpentier E. A programmable dual-RNA–guided DNA endonuclease in adaptive bacterial immunity. Science. 2012;337: 816–821. 10.1126/science.1225829 22745249 PMC6286148

[6] Cong L, Ran FA, Cox D, Lin S, Barretto R, Habib N, et al Multiplex genome engineering using CRISPR/Cas systems. Science. 2013;339: 819–823. 10.1126/science.1231143 23287718 PMC3795411

[7] Xue W, Chen S, Yin H, Tammela T, Papagiannakopoulos T, Joshi NS, et al CRISPR-mediated direct mutation of cancer genes in the mouse liver. Nature. 2014;514: 380–384. 10.1038/nature13589 25119044 PMC4199937

[8] Ran FA, Hsu PD, Lin CY, Gootenberg JS, Konermann S, Trevino AE, et al Double nicking by RNA-guided CRISPR Cas9 for enhanced genome editing specificity. Cell. 2013;154: 1380–1389. 10.1016/j.cell.2013.08.021 23992846 PMC3856256

[9] Zhou X, Xin J, Fan N, Zou Q, Huang J, Ouyang Z, et al Generation of CRISPR/Cas9-mediated gene-targeted pigs via somatic cell nuclear transfer. Cell Mol Life Sci. 2015;72: 1175–1184. 10.1007/s00018-014-1744-7 25274063 PMC11113635

[10] Ni W, Qiao J, Hu S, Zhao X, Regouski M, Yang M, et al Efficient gene knockout in goats using CRISPR/Cas9 system. PLoS One. 2014;9: e106718 10.1371/journal.pone.0106718 25188313 PMC4154755

[11] Song Y, Yuan L, Wang Y, Chen M, Deng J, Lv Q, et al Efficient dual sgRNA-directed large gene deletion in rabbit with CRISPR/Cas9 system. Cell Mol Life Sci. 2016;73: 2959–2968. 10.1007/s00018-016-2143-z 26817461 PMC11108552

[12] Hwang WY, Fu Y, Reyon D, Maeder ML, Tsai SQ, Sander JD, et al Efficient in vivo genome editing using RNA-guided nucleases. Nat Biotechnol. 2013;31: 227–229. 10.1038/nbt.2501 23360964 PMC3686313

[13] Zhou H, Liu B, Weeks DP, Spalding MH, Yang B. Large chromosomal deletions and heritable small genetic changes induced by CRISPR/Cas9 in rice. Nucleic Acids Res. 2014;42: 10903–10914. 10.1093/nar/gku806 25200087 PMC4176183

[14] Xing H-L, Dong L, Wang Z-P, Zhang H-Y, Han C-Y, Liu B, et al A CRISPR/Cas9 toolkit for multiplex genome editing in plants. BMC Plant Biol. 2014;14: 327 10.1186/s12870-014-0327-y 25432517 PMC4262988

[15] Bai Y, He L, Li P, Xu K, Shao S, Ren C, et al Efficient genome editing in chicken DF-1 cells using the CRISPR/Cas9 system. G3 (Bethesda). 2016;6: 917–923. 26869617 10.1534/g3.116.027706 PMC4825661

[16] Véron N, Qu Z, Kipen PAS, Hirst CE, Marcelle C. CRISPR mediated somatic cell genome engineering in the chicken. Dev Biol. 2015;407: 68–74. 10.1016/j.ydbio.2015.08.007 26277216

[17] Oishi I, Yoshii K, Miyahara D, Kagami H, Tagami T. Targeted mutagenesis in chicken using CRISPR/Cas9 system. Sci Rep. 2016;6: 23980 10.1038/srep23980 27050479 PMC4822141

[18] Zuo Q, Wang Y, Cheng S, Lian C, Tang B, Wang F, et al Site-directed genome knockout in chicken cell line and embryos can use CRISPR/Cas gene editing technology. G3 (Bethesda). 2016;6: 1787–1792. 27172204 10.1534/g3.116.028803 PMC4889674

[19] Dimitrov L, Pedersen D, Ching KH, Yi H, Collarini EJ, Izquierdo S, et al Germline gene editing in chickens by efficient CRISPR-mediated homologous recombination in primordial germ cells. PLoS One. 2016;11: e0154303 10.1371/journal.pone.0154303 27099923 PMC4839619

[20] Kagawa W, Arai N, Ichikawa Y, Saito K, Sugiyama S, Saotome M, et al Functional analyses of the C-terminal half of the *Saccharomyces cerevisiae* Rad52 protein. Nucleic Acids Res. 2014;42: 941–951. 10.1093/nar/gkt986 24163251 PMC3902949

[21] Di Primio C, Galli A, Cervelli T, Zoppe M, Rainaldi G. Potentiation of gene targeting in human cells by expression of *Saccharomyces cerevisiae* Rad52. Nucleic Acids Res. 2005;33: 4639–4648. 10.1093/nar/gki778 16106043 PMC1187822

[22] Kalvala A, Rainaldi G, Di Primio C, Liverani V, Falaschi A, Galli A. Enhancement of gene targeting in human cells by intranuclear permeation of the *Saccharomyces cerevisiae* Rad52 protein. Nucleic Acids Res. 2010;38: e149 10.1093/nar/gkq486 20519199 PMC2919737

[23] Ren C, Xu K, Liu Z, Shen J, Han F, Chen Z, et al Dual-reporter surrogate systems for efficient enrichment of genetically modified cells. Cell Mol Life Sci. 2015;72: 2763–2772. 10.1007/s00018-015-1874-6 25725802 PMC11113981

[24] Johnson S, Nguyen V, Coder D. Assessment of cell viability. Curr Protoc Cytom. 2013;64: 9.2.1–9.2.26. 10.1002/0471142956.cy0902s64 23546778

[25] Stinckens A, Georges M, Buys N. Mutations in the Myostatin gene leading to hypermuscularity in mammals: indications for a similar mechanism in fish? Anim Genet. 2011;42: 229–234. 10.1111/j.1365-2052.2010.02144.x 21175702

[26] Grobet L, Martin LJ, Poncelet D, Pirottin D, Brouwers B, Riquet J, et al A deletion in the bovine myostatin gene causes the double-muscled phenotype in cattle. Nat Genet. 1997;17: 71–74. 10.1038/ng0997-71 9288100

[27] Clop A, Marcq F, Takeda H, Pirottin D, Tordoir X, Bibe B, et al A mutation creating a potential illegitimate microRNA target site in the myostatin gene affects muscularity in sheep. Nat Genet. 2006;38: 813–818. 10.1038/ng1810 16751773

[28] Qian L, Tang M, Yang J, Wang Q, Cai C, Jiang S, et al Targeted mutations in myostatin by zinc-finger nucleases result in double-muscled phenotype in Meishan pigs. Sci Rep. 2015;5: 14435 10.1038/srep14435 26400270 PMC4585837

[29] Crispo M, Mulet AP, Tesson L, Barrera N, Cuadro F, dos Santos-Neto PC, et al Efficient generation of myostatin knock-out sheep using CRISPR/Cas9 technology and microinjection into zygotes. PLoS One. 2015;10: e0136690 10.1371/journal.pone.0136690 26305800 PMC4549068

[30] Zhang GX, Zhao XH, Wang JY, Ding FX, Zhang L. Effect of an exon 1 mutation in the myostatin gene on the growth traits of the Bian chicken. Anim Genet. 2012;43: 458–459. 10.1111/j.1365-2052.2011.02274.x 22497311

[31] Kim H, Um E, Cho SR, Jung C, Kim H, Kim JS. Surrogate reporters for enrichment of cells with nuclease-induced mutations. Nat Methods. 2011;8: 941–943. 10.1038/nmeth.1733 21983922

[32] Kim H, Kim MS, Wee G, Lee CI, Kim H, Kim JS. Magnetic separation and antibiotics selection enable enrichment of cells with ZFN/TALEN-induced mutations. PLoS One. 2013;8: e56476 10.1371/journal.pone.0056476 23441197 PMC3575389

[33] Ramakrishna S, Cho SW, Kim S, Song M, Gopalappa R, Kim JS, et al Surrogate reporter-based enrichment of cells containing RNA-guided Cas9 nuclease-induced mutations. Nat Commun. 2014;5: 3378 10.1038/ncomms4378 24569644

[34] Moehle EA, Rock JM, Lee Y-L, Jouvenot Y, DeKelver RC, Gregory PD, et al Targeted gene addition into a specified location in the human genome using designed zinc finger nucleases. Proc Natl Acad Sci U S A. 2007;104: 3055–3060. 10.1073/pnas.0611478104 17360608 PMC1802009

[35] Chen F, Pruett-Miller SM, Huang Y, Gjoka M, Duda K, Taunton J, et al High-frequency genome editing using ssDNA oligonucleotides with zinc-finger nucleases. Nat Methods. 2011;8: 753–755. 10.1038/nmeth.1653 21765410 PMC3617923

[36] Inui M, Miyado M, Igarashi M, Tamano M, Kubo A, Yamashita S, et al Rapid generation of mouse models with defined point mutations by the CRISPR/Cas9 system. Sci Rep. 2014;4: 5396 10.1038/srep05396 24953798 PMC4066261

[37] Sauer NJ, Narvaez-Vasquez J, Mozoruk J, Miller RB, Warburg ZJ, Woodward MJ, et al Oligonucleotide-mediated genome editing provides precision and function to engineered nucleases and antibiotics in plants. Plant Physiol. 2016;170: 1917–1928. 10.1104/pp.15.01696 26864017 PMC4825113

[38] Xia SJ, Shammas MA, Shmookler Reis RJ. Elevated recombination in immortal human cells is mediated by HsRAD51 recombinase. Mol Cell Biol. 1997;17: 7151–7158. 9372947 10.1128/mcb.17.12.7151 PMC232572

[39] Sugiyama T, Kantake N. Dynamic regulatory interactions of Rad51, Rad52, and replication protein-A in recombination intermediates. J Mol Biol. 2009;390: 45–55. 10.1016/j.jmb.2009.05.009 19445949

[40] Sacco MA, Nair VK. Prototype endogenous avian retroviruses of the genus *Gallus*. J Gen Virol. 2014;95: 2060–2070. 10.1099/vir.0.066852-0 24903328

[41] Sacco MA, Flannery DM, Howes K, Venugopal K. Avian endogenous retrovirus EAV-HP shares regions of identity with avian leukosis virus subgroup J and the avian retrotransposon ART-CH. J Virol. 2000;74: 1296–1306. 10627540 10.1128/jvi.74.3.1296-1306.2000 PMC111464

[42] Wragg D, Mason AS, Yu L, Kuo R, Lawal RA, Desta TT, et al Genome-wide analysis reveals the extent of EAV-HP integration in domestic chicken. BMC Genomics. 2015;16: 784 10.1186/s12864-015-1954-x 26466991 PMC4607243

