# Supplementary figures and images for: Enhancing Targeted Genomic DNA Editing in Chicken Cells Using the CRISPR/Cas9 System
Source: PLoS One. 2017 Jan 9;12(1):e0169768. doi: 10.1371/journal.pone.0169768 (PMC5222187; doi:10.1371/journal.pone.0169768)

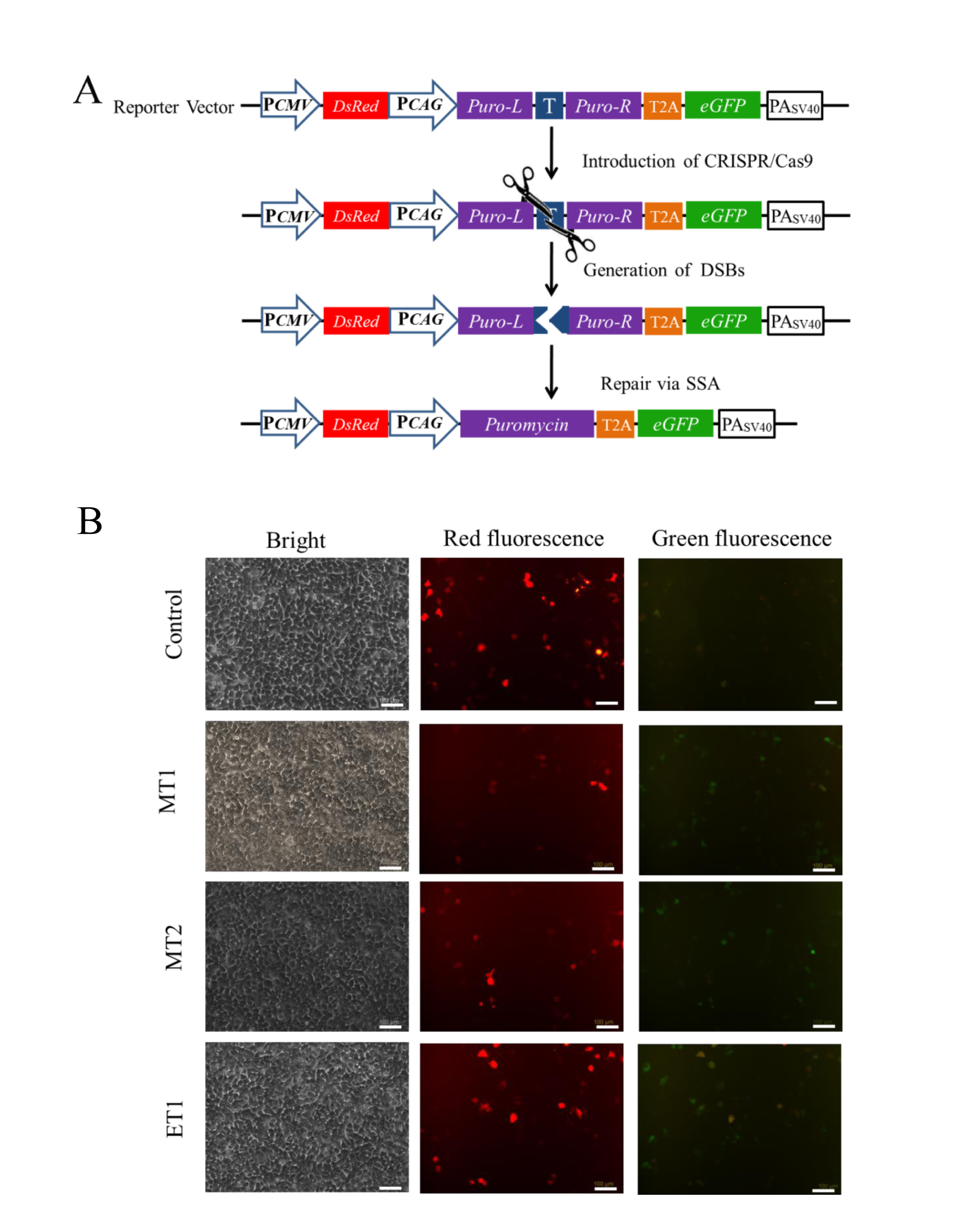

Supplement: S1 Fig — (A) A schematic diagram of the dual reporter surrogate system. Under the control of the CMV promoter, the expression of DsRed was directly detectable after transfection into HEK293T cells. Two direct repeats and target sequences with PAM divided the puromycin resistance gene. A customized CRISPR/Cas9 cut at the target sites in the reporter vector to generate DSBs, which were repaired via single strand annealing in the presence of homologous arms. Subsequently, the wild-type puromycin resistance (puroR) gene was restored, as was functional eGFP. Thus, puroR and eGFP, as dual reporter genes, were used for CRISPR/Cas9 activity validation and gene-editing positive colony screening. (B) Validating the targeted cleavage of designed CRISPR/Cas9 in HEK293T cells. CRISPR/Cas9 expression vectors and their corresponding reporter vectors at the MT1, MT2 and ET1 sites were co-transfected into HEK293T cells, respectively. Cells transfected with empty expression and nonsense reporter vectors were used as controls. RFP-positive or GFP-positive cells were observed via fluorescence microscopy. Scale bar = 200 μm. (TIF) [file pone.0169768.s001.tif]

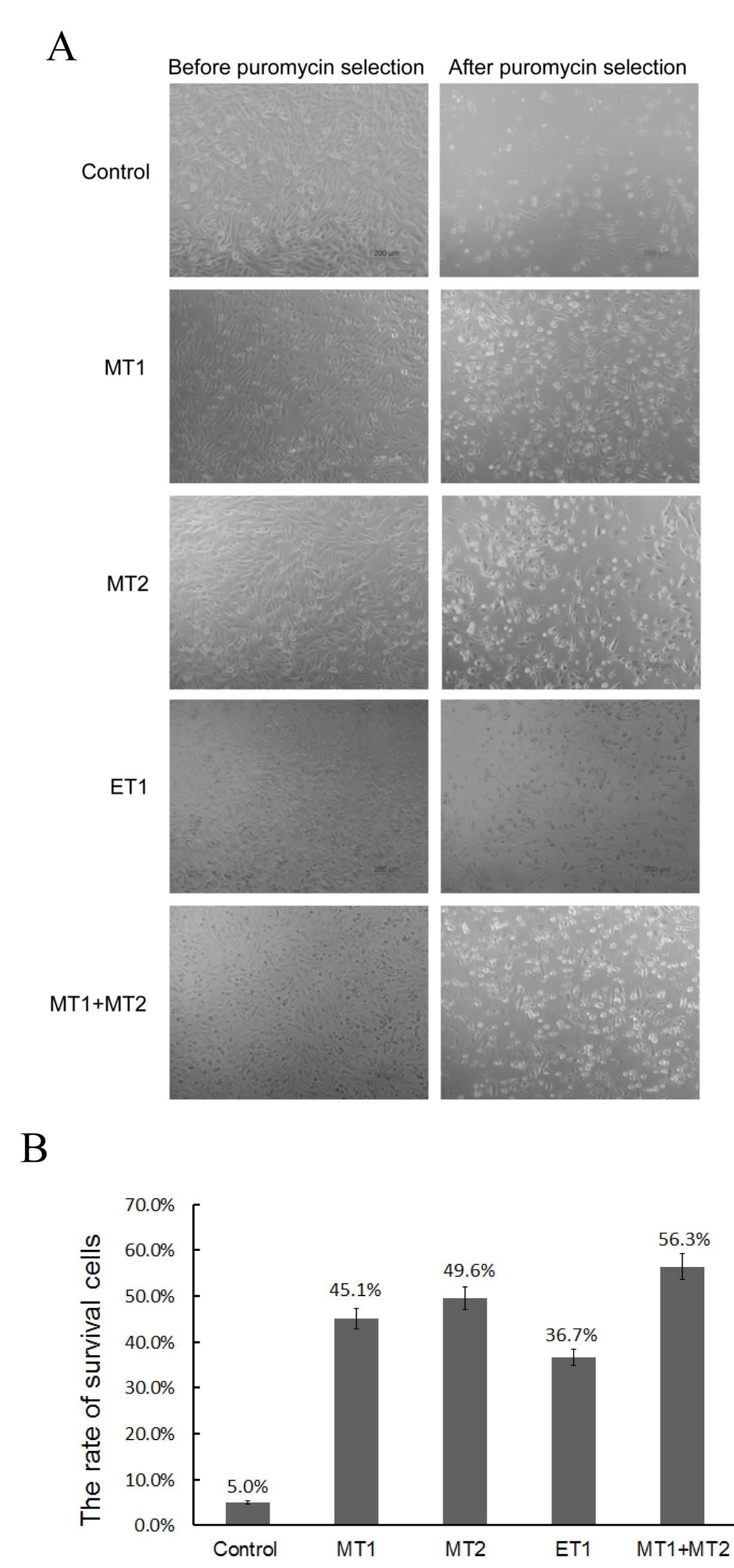

Supplement: S2 Fig — DF-1 cells were co-transfected with CRISPR/Cas9 expression and their corresponding vectors. Puromycin was added to the cell culture medium after the 48-h transformation at a final concentration of 2.5 μg/mL. After selection for 4 days, cells were observed via microscopy, and the rates of survival were determined via flow cytometry. Cells transfected with empty CRISPR/Cas9 expression vector and reporter plasmid without target sites were used as controls. MT1, MT2 and ET1 indicate the related experimental groups of cells treated with vectors for the single target site. MT1+MT2 indicate cells treated with vectors for both the MT1 and MT2 target sites. After puromycin selection, the cells in each well were divided into two groups. (A) One group was maintained in fresh medium for observation via microscopy. Scale bar = 200 μm. (B) The other group was used to calculate the survival rate via PI staining. (TIF) [file pone.0169768.s002.tif]

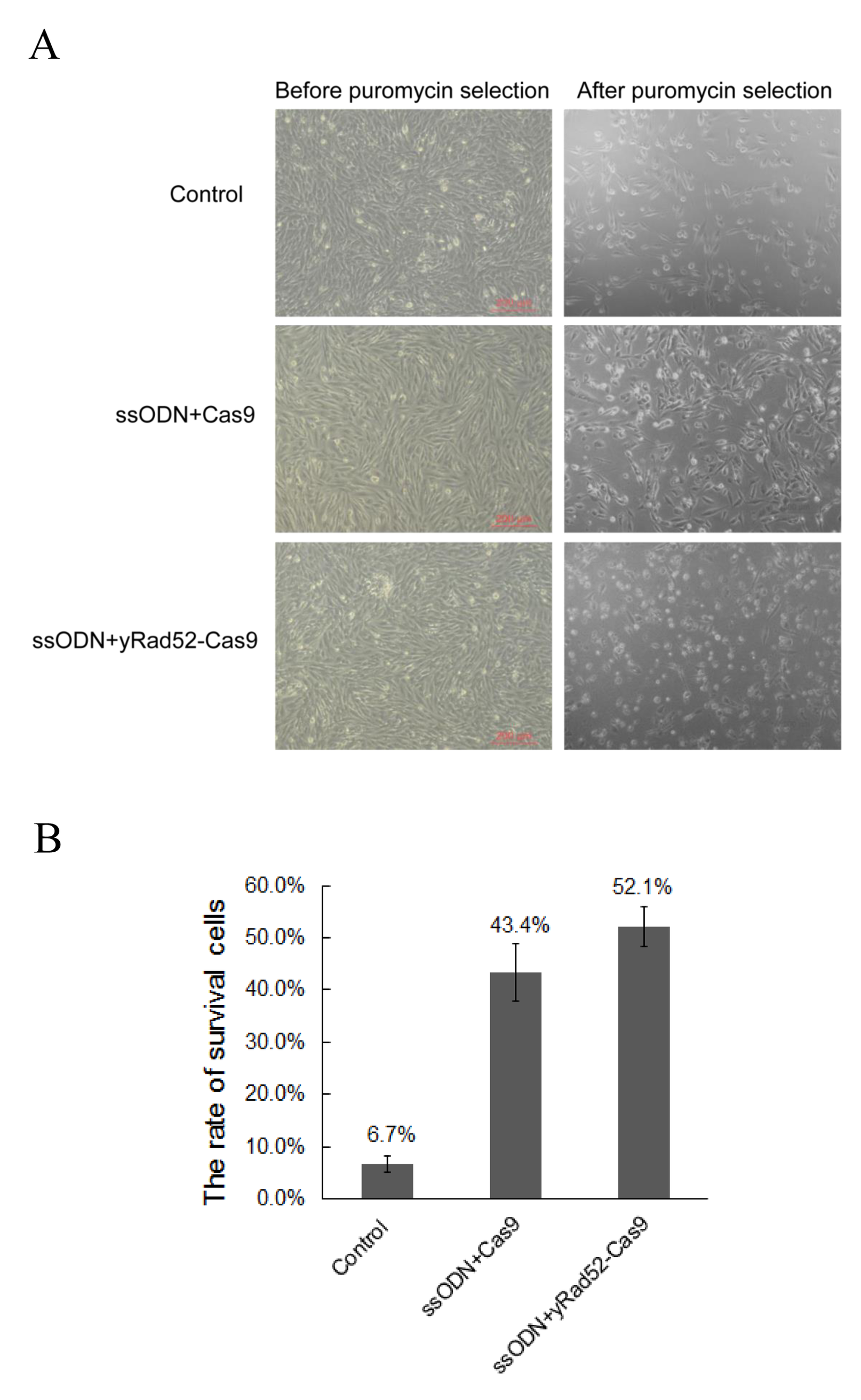

Supplement: S3 Fig — Cells transfected with 0.5 nmol of ssODN were used as controls. Cells treated with 0.5 nmol ssODN, 1 μg of MT1 reporter vector and 1 μg of MT1-CRISPR/Cas9 or CRISPR/yRad52-Cas9 expression vector were used as experimental groups 2 and 4. After transfection for 2 days, cells were maintained in medium with puromycin (2.5 μg/mL) for 4 days. After puromycin selection, cells were divided into two groups. (A) One group of cells was grown in fresh medium without puromycin for 2–3 days. The cells were then observed via microscopy. Scale bar = 200 μm. (B) The other cells were used to calculate the survival rate via PI staining. (TIF) [file pone.0169768.s003.tif]

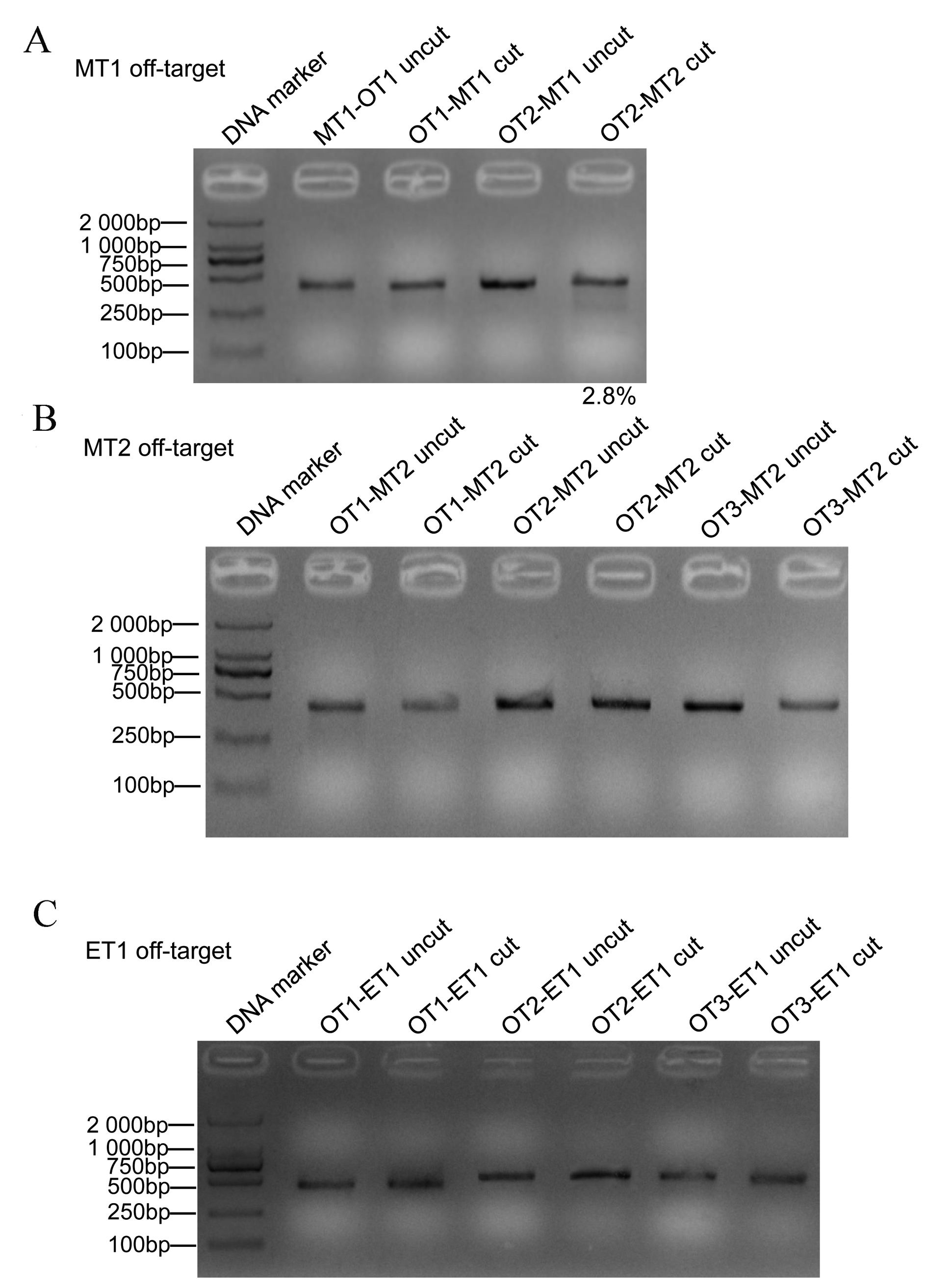

Supplement: S4 Fig — (A) The off-target effect of the two candidates for the MT1 target site. (B) The off-target effect of the three candidates for the MT2 target site. (C) The off-target effect of the three candidates for the ET1 target site. (TIF) [file pone.0169768.s004.tif]
