# Supplementary material for: Enhancing Targeted Genomic DNA Editing in Chicken Cells Using the CRISPR/Cas9 System
Source: PLoS One. 2017 Jan 9;12(1):e0169768. doi: 10.1371/journal.pone.0169768 (PMC5222187; doi:10.1371/journal.pone.0169768)
Supplement: S1 Text — (DOC) [file pone.0169768.s005.doc]

**S1 Text Primers for vector construction and detection of targeted modification**

Table A primers for CRISPR/Cas9 expression and reporter vectors construction

| Primer name | Sequences (5’- 3’) | Note |
| --- | --- | --- |
| U6F | CGCTCTAGATTTCCCATGATTCCTTCATAT | Universal primers for sgRNA construction |
| U6R | ACCGCGGCCGCAAAAAAGCACCGACTCGGTGCC |
| MT1F | GATAACGTCCCTGCTAATGTT GTTTTAGAGCTAGAAATAGCAAG | MT1 sgRNA construction |
| MT1R | AACATTAGCAGGGACGTTATC GGTGTTTCGTCCTTTCCACAAG |
| MT2F | GCTGTCACATTCCCAGGACC GTTTTAGAGCTAGAAATAGCAAG | MT2 sgRNA construction |
| MT2R | GGTCCTGGGAATGTGACAGC GGTGTTTCGTCCTTTCCACAAG |
| ET1F | GTACTCCTGGCGCAGGGACAC GTTTTAGAGCTAGAAATAGCAAG | ET1 sgRNA construction |
| ET1R | GTGTCCCTGCGCCAGGAGTAC GGTGTTTCGTCCTTTCCACAAG |
| MT1-ReF | ggccgcATAACGTCCCTGCTAATGTTAGGg | MT1 reporter construction |
| MT1-ReR | gatccCACCTAACATTAGCAGGGACGTTATgc |
| MT2-ReF | ggccgc GCTGTCACATTCCCAGGACCGGGg | MT2 reporter construction |
| MT2-ReR | gatcc CACCCGGTCCTGGGAATGTGACAGCgc |
| ET1-ReF | ggccgc TACTCCTGGCGCAGGGACACGGGg | ET1 reporter construction |
| ET1-ReR | gatccCCC GTGTCCCTGCGCCAGGAGTA gc |
| yRadF | cccGGTCTCACTAGAATGAATGAAATTATGGATATGGATG | Amplification of yeast Rad52 |
| yRadR | gggGGTCTCAGATCGAGTAGGCTTGCGTGCATGCAG |

Note: Letters with underline were target sequences, and letters with double underline were PAM sequences. Lowcase letters stand for sticky end for BamHI and NotI. Letters with doule-under line were recognition sites for BsaI.

Table B Primers for donor DNA construction

| Primer name | Sequences (5’- 3’) | Note |
| --- | --- | --- |
| ArmL-F | ccc AAGCTT TAGGACAATTGACTATGCTCTC | Amplification of 300 bp left homology arm |
| ArmL-R | GTGTCCTCCAGCATCGCATTCA |
| ArmR-F | GGGATGTGTTGCTTCAATCTCA | Amplification of 200 bp right homology arm |
| ArmR-R | cg GAATTC GAAAGCACTTGATTATACATGG |
| L-EGFPF | TGAATGCGATGCTGGAGGACAC TAGTAATCAATTACGGGGTC | Amplification of EGFP expression cassette |
| R-EGFPR | TGAGATTGAAGCAACACATCCC GATACATTGATGAGTTTGGACA |
| ssODN | CAGCAAACTGCGGCTGGAACAAGCACCTAACGAATTCAGGGACGTTATTA | Donor DNA for MT1 site |

Note: Letters with underline were EcoRI restriction site.

Table C Primers for amplifying target sequences

| Primer name | Sequence (5’- 3’) | Product size |
| --- | --- | --- |
| MT1PF | GCTGTCACGGATCTGTTTAG | 489bp |
| MT1PR | AGACGAAAGCAGCAGGGTTG |
| MT2PF | GATATTGCATCCACTCTGTTACC | 626bp |
| MT2PR | ACCTTCATCTGCCATTCTCG |
| ET1PF | CTCTCTCCTAACTTTACCACCTG | 516bp |
| ET1PR | CTCTCTCCTAACTTTACCACCT |
| EPF | ATTGGTGTAGTTCAAAAAGGGG | 1200bp |
| EGFPR | ACTTGTGGCCGTTTACGTCG |
