# Supplementary material for: Enhancing Targeted Genomic DNA Editing in Chicken Cells Using the CRISPR/Cas9 System
Source: PLoS One. 2017 Jan 9;12(1):e0169768. doi: 10.1371/journal.pone.0169768 (PMC5222187; doi:10.1371/journal.pone.0169768)
Supplement: S2 Text — (DOC) [file pone.0169768.s006.doc]

**S2 Text Potential off target sites and their corresponding primers**

Table A Candidates of off target sequences

| Off-target sites | Sequences (5’- 3’) |
| --- | --- |
| MT1-OT1 | ATgAaGaCCtTGCTAATGTT CAG |
| MT1-OT2 | AcAAtGTagCTGCTAATGTT AAG |
| MT2-OT1 | GtTGaCAgATTCCCAGGACC TGG |
| MT2-OT2 | caTcTCACAcTCCCAGGACC TAG |
| MT2-OT3 | GtgGaCACATTCCCAGtACC AAG |
| ET1-OT1 | TcCTCCTGGcGCAGGGACaC TGG |
| ET1-OT2 | cACTtCTGaCgCAGGGACAC TGG |
| ET1-OT3 | TtgTCCTtGtGCAGGGACAC TAG |

Note: Lowercase letters indicated nucleotides in off-target sequences mismatched with target sites. Nucleotides with underline were PAM sequences for off-target candidates.

Table B Primers for amplifying off-target sequences

| Primer name | Sequence (5’- 3’) | Product size |
| --- | --- | --- |
| MT1-OT1F | GGGCTCTTGAGACTGGTTTATGA | 420bp |
| MT1-OT1R | AGTACAGCAACGAGCAACCTCTT |
| MT1-OT2F | GTAGTGATTTCGATTTTCATACAGG | 429bp |
| MT1-OT2R | ATAAGAACCGCAGTCTCTCCAG |
| MT2-OT1F | AAACTTCCTGACTCTAACACGA | 440bp |
| MT2-OT1R | GCTGTAAATGTAGAATTTGCTG |
| MT2-OT2F | TGTGTGTTCACTGGCTCCACGAG | 440bp |
| MT2-OT2R | TGGCCTTTGTCATTCAGCTTCTG |
| MT2-OT3F | GGGCTCTTGAGACTGGTTTATGA | 420bp |
| MT2-OT3R | AGTACAGCAACGAGCAACCTCTT |
| ET1-OT1F | CAGGAGCCCACTAGAGAAGAGA | 442bp |
| ET1-OT1R | TGAAGGTCAGGCAGACAAAAGA |
| ET1-OT2F | TGCGAATGAAAGGCAGTAGGAG | 496bp |
| ET1-OT2R | GTCACGGCAGTGGATAAAAGGT |
| ET1-OT3F | TTCTCCCACTGTCAAGTAGGTC | 552bp |
| ET1-OT3R | TTCCATCTTCCCATGTTGTATC |
